# Structural Factors and Quality of Diabetes Health Services in Hail, Saudi Arabia: A Cross-Sectional Study

**DOI:** 10.3390/healthcare9121691

**Published:** 2021-12-07

**Authors:** Ramaiah Itumalla, Rakesh Kumar, Mohamed Tharwat Elabbasy, Bilesha Perera, Mohammad R. Torabi

**Affiliations:** 1 Department of Health Management, College of Public Health and Health Informatics, University of Hail, Hail 81451, Saudi Arabia; r.itumalla@uoh.edu.sa (R.I.); bileshap@gmail.com (B.P.); 2 Department of Public Health, College of Public Health and Health Informatics, University of Hail, Hail 81451, Saudi Arabia; tharwat330@gmail.com; 3 Faculty of Medicine, University of Ruhuna, Galle 80000, Sri Lanka; 4 School of Public Health, Indiana University, Bloomington, IN 47405, USA; torabi@indiana.edu

**Keywords:** diabetes, structural factors, prevention, Hail, Saudi Arabia

## Abstract

The chronic disease burden in Saudi Arabia has created adverse health, social and economic consequences that require urgent attention from health and political authorities. Diabetes has become an epidemic in Saudi Arabia. Data on personal and structural factors associated with diabetes in the Hail region are scarce. Such data are imperative to develop effective strategies to control the epidemic in the region. A cross-sectional study of diabetes patients attending diabetes health care facilities in Hail was conducted using a sample of 392 patients. An interviewer-administered questionnaire was used. A slightly higher proportion of female participants (54.1%) were included in the sample. Most of the participants were from rural areas (73.9%), and 70.9% of the participants were from the middle-age (30–50 years) category. A close proximity to the diabetes clinic (OR = 1.98; 95% CI: 1.08–3.44), good transport facilities (OR = 1.67; 95% CI: 1.11–2.78) and feeling contented with supportive services (OR = 2.03; 95% CI: 1.12–4.04) were associated with patients’ satisfaction with the overall quality of the diabetes clinic services. The presence of good-quality health care professionals working in these treatment centers also seemed to contribute to patients’ satisfaction with the services they received. These structural factors associated with patients’ satisfaction with the services they received from diabetes clinics must be considered in diabetes control programs in the region. The minimization of structural barriers will eventually assist the national strategic plan, Vision 2030, which aims to improve the quality of life of the Saudi people by 2030.

## 1. Introduction

Diabetes has become a worldwide epidemic, and many countries around the world are struggling to overcome the health and economic burdens associated with it [1,2]. Diabetes is a chronic non-communicable disease (NCD) caused by a malfunction in the formation of insulin in the pancreas or by the ineffective use of insulin produced by the body [3,4]. These irregularities in the production and use of insulin in the body elevate the blood sugar (or blood glucose) levels, causing serious damages to many parts in the body, including the heart, eyes, kidneys and nerves [3]. There are two types of diabetes: type 1 and type 2. Type 1 diabetes (T1D), of which the cause is unknown, is seen most frequently in children and adolescents. Type 2 diabetes (T2D), which is caused by the body’s ineffective use of insulin, is the most common type seen in older adults. Type 2 diabetes accounts for approximately 90% of all diabetes cases around the world [4]. Globally, an estimated 449 million adults (aged 20–99 years) lived with diabetes in 2017, a fourfold increase from 1980 [5], and the number of cases is expected to exceed 552 million by 2030. Since diabetes causes a significant economic burden on health care systems and on families of diabetes patients [4,5], the increasing trends of incidence and prevalence of diabetes seen in many countries would be a devastating factor to the economic development of such countries. In addition to the economic burden on the health care system and national economy, diabetes would also impose a large economic burden to families with diabetic patients in terms of higher out-of-pocket health care payments and the loss of family income associated with disability, premature death and caring for disabled family members. Expenditure on medications and treatments is a major household expenditure in such families. 

The World Health Organization (WHO) revealed that Saudi Arabia ranks the second highest in the Middle East and seventh in the world for the rate of diabetes [5,6,7,8]. Today, there are approximately seven million diabetic cases and three million cases of pre-diabetics in Saudi Arabia. Data indicate that the prevalence of T2D among the Saudi population is increasing dramatically. Thus, diabetes has become one of the most challenging chronic health problems faced by people in Saudi Arabia.

This increasing trend of diabetes observed in Saudi Arabia can be attributed to various factors. Escalating trends of the obesity rate in the country, poor food habits, and population aging are the three main reasons for this trend [6,8]. A healthy diet, physically active lifestyle and weight control have been identified as preventive strategies in controlling diabetes in Saudi Arabia. In addition to these personal attributes, health care system deficiencies, environmental barriers to accessing treatment and medical advice, and cultural beliefs on alternative and allopathic treatments may also have significantly contributed to the increasing prevalence and incidence of diabetes in Saudi Arabia [6,9,10,11]. A multidisciplinary approach is required to achieve the desired outcomes related to diabetes control in the country. However, when compared with the research conducted in the high-income countries, research on the epidemiological underpinnings, health education and health promotion of diabetes conducted in Saudi Arabia is grossly inadequate. Studies have revealed that the level of knowledge of the disease among diabetes patients in Saudi Arabia is somewhat low [8,10], making Saudi people more vulnerable to T2D. The government of Saudi Arabia has focused on controlling the epidemic using educational, screening and treatment interventions. The government’s national strategic plan, Vision 2030, has given high priority to controlling the chronic diseases prevalent in the country [12]. This study is aimed at identifying the personal and structural factors associated with diabetes among people in Hail, Saudi Arabia. We hypothesized that a long distance to health care centers, long waiting hours, poor transport facilities, deficiencies in paramedical services and the poor service quality provided by health care workers would jeopardize patients motivation to use available diabetes health care services in Hail. The findings will be highly useful to raise awareness of the vital roles played by personal and structural factors related to diabetes prevention in Saudi Arabia among the public and health policymakers. 

## 2. Materials and Methods

### 2.1. Study Design and Sampling

This was a cross-sectional study. Participants were recruited from diabetes clinics in several health care centers in Hail. Diabetes patients using government health care facilities were the target population. There are both public and private secondary and tertiary care facilities in Hail. In addition, diabetes clinics are run by primary health care centers (PHCC) in the country. However, as seen in many developed countries, physician-led community nursing services are simply not available in Hail. We randomly selected 16 diabetes clinics operated in Hail. From those selected centers, 25 patients from each center were selected using a systematic sampling method. Those who had provided informed consent were interviewed using an interviewer-administrated questionnaire. A total of 400 patients were initially selected for the survey. An interviewer-administered questionnaire was prepared using a literature survey. 

### 2.2. The Measurements

An interviewer-administered questionnaire was used. The questionnaire consisted of three sections. Section 1 contained questions on patient demographic details, including age, sex, marital status and educational level. Section 2 contained a series of questions on the general health status, family support and hospital environment. Section 3 consisted of questions related to structural factors associated with controlling diabetes in the target population. The structural factors investigated in this study included the distance to the diabetes clinic; the waiting time in the clinic; transport facilities; the availability of other supportive services, such as counselling, health education, support for disabled patients, etc.; and the service quality of doctors, nurses, pharmacists, and medical laboratory personnel as perceived by the patients. Participants’ satisfaction of the services provided by the health care professionals and the overall satisfaction of the services provided by the clinic were assessed using a scale of 1 to 10, where 1 indicates “highly dissatisfied” and 10 indicates “highly satisfied”. Those who reported scores of or above six were categorized as satisfied with the services, while the others were categorized as not satisfied with the services.

### 2.3. Data Collection

All the participants were educated about the questionnaire in the relevant clinic wards before administrating the questionnaire. Participants were given the opportunity to ask any questions concerning the questionnaire, consent for the study, and the purpose and benefits of the study. Participants were enrolled on a voluntary basis. All participants were informed about the usage of data of the study. The confidentiality of the data was ensured for all information collected, and no identification information was collected from the participants. Data were presented in aggregated terms. 

### 2.4. Ethical Considerations

Ethical approval for the study was obtained from the Ethical Review Committee, University of Hail Permission number: H-2020-197). Permission was obtained from the person in charge of the selected health care facilities. 

### 2.5. Data Analysis

All data were coded and entered into a database, which was created using the Statistical Package of Social Science (SPSS). Data cleaning and checking were performed before analysis. The answers from the questionnaire were coded, and marks were allocated accordingly. Where appropriate, data were expressed as means and standard deviations or as proportions. Differences between groups were tested for statistical significance using the chi-squared test and *t*-test. Significance was considered as *p* < 0.05. 

## 3. Results

A total of 392 participants who provided consent for the survey were included in the analysis. Eight participants were excluded from the analysis due to incomplete answers. Table 1 summarizes the demographic characteristics of the participants. In total, 180 (45.9%) men and 212 (54.1%) women were included in the study. The majority (70.9%) were middle-aged people (aged between 30 and 50 years). A total of 310 (79.1%) participants were married, and 73.9% (*n* = 290) were from rural areas. Nearly 83% of the participants reported that their family monthly income was less than SR 10,000. About 71% of the participants were engaged in either government or private sector jobs at the time of the survey. 

Among men, 47.2% (*n* = 85) were satisfied with the overall services provided by the facility, and while 50.9% (*n* = 108) of women were satisfied. No significant gender difference was observed. The distance from home to the diabetes center was a factor determining the quality of services perceived by the participants (Table 2). Patients who had less than 2 km distance from their home to their clinic were more likely to report satisfaction with the overall quality of the services provided by the center (OR = 1.98; 95% CI: 1.08–3.44). No significant association was observed between the waiting time and overall satisfaction of the services obtained. Those who were satisfied with their transport facilities were more likely to report overall satisfaction with the services provided by the center (OR = 1.67; 95% CI: 1.11–2.78). Those who were satisfied with other supportive services at the health care center were more likely to report overall satisfaction with the services provided by the center (OR = 2.03; 95% CI: 1.12–4.04).

Those who were satisfied with the services provided by the doctors (OR = 2.54; 95% CI: 1.21–3.98), pharmacists (OR = 1.66; 95% CI: 1.06–2.17) and medical lab personnel (OR = 1.56; 95% CI: 1.03–2.44 in the center were more likely to report overall satisfaction with the services provided by the center. No significant association was found between the service quality of nurses and overall satisfaction with the services provided by the center.

## 4. Discussion

Vision 2030, the national strategic plan to achieve health and economic prosperity in Saudi Arabia by 2030, has recognized the elimination of health disparities in patients with chronic diseases as a public health priority [12,13]. Diabetes is an important chronic disease condition targeted by this initiative. Enhancements of the acceptability and accessibility of preventive and curative health care services for diabetes patients in the country are considered as main strategies in preventing and controlling diabetes in Saudi Arabia. Personal risk factors of diabetes, such as poor eating habits, sedentary lifestyles, advanced age, poverty and smoking, have been studied extensively across the world, as well as in Saudi Arabia [8,14]. However, less attention has been paid to accessing and designing strategies to overcome the structural and cultural barriers associated with diabetes in Saudi Arabia. The results of this study shed light in areas of diabetes control that can be considered vital to achieve the objectives of Vision 2030. 

In this study, we found that a close proximity to the diabetes clinic would increase patients’ satisfaction with services they expect from the clinics. A study conducted in Canada revealed that access to care closer to home may benefit glycemic control in children with T1D and improve treatment satisfaction [15]. Another study conducted in Haiti showed that distance from the clinic was associated with visit adherence measures among patients during their clinic visits in the last quarter [16]. It is possible to eliminate long distances to health care services by increasing the number of diabetes clinics in rural areas and by introducing mobile health care services. Such facilities would motivate patients to use available health care resources in an effective way and to increase drug adherence rates. This would eventually minimize unmet needs. 

The non-availability or inadequacy of transport was found to be a barrier to satisfaction with clinical services received by the respondents. This finding is in line with similar studies conducted in other countries. A study conducted in Ethiopia showed that cost of transport to the hospital was significantly associated with non-adherence to the diabetic treatment regimen [17]. Specifically, poor patients in rural areas face limited transport facilities, and health authorities need to plan a strategy, such as introducing public transport options for clinical patients by using a multi-sectoral approach. 

Lack of awareness of the disease and inadequate glycemic control may put patients in danger. There is increasing evidence that patient education, proper counselling and good nutrition are the most effective ways to lessen the complications of diabetes [18,19]. Compared with the lack of a formal education, greater educational attainment was associated with an increased risk of diabetes and cardiovascular diseases [20,21]. However, the level of education is not a barrier for changing unhealthy lifestyle patterns found in middle and upper income countries [22]. Our findings also indicate that diabetes patients who were satisfied with other supportive services have a greater likelihood of being satisfied with the overall service quality of diabetes clinics. This would eventually help the patients adhere to medical advice and to lower their health complications. Health authorities in Hail should consider enhancing in-service training for the paramedical staff attached to diabetic clinics to educate patients and to provide necessary paramedical services.

In this study, the participants who were satisfied with the quality of services provided by health professionals were more likely to report greater satisfaction with diabetes health care. Patient satisfaction is recognized as an important indicator for quality of care and adherence rates. Good personal characteristics and communication skills of health care professionals are associated with higher levels of patient satisfaction [23,24,25]. However, further research on the cultural and religious aspects of health care services is warranted in this area to confirm our hypotheses.

## 5. Conclusions

Diabetes is a serious and complicated public health issue in Saudi Arabia. The Saudi government is keen to provide infrastructure and health care support in controlling this epidemic. Increasing patient awareness of the disease and its complications and motivating them to adhere to treatment regimens are necessary to control this public health issue. In this endeavor, structural barriers, such as long distances to treatment facilities, transport difficulties, deficiencies in supportive services and the low quality of services rendered by health care professionals require urgent attention from health care authorities and policymakers in Saudi Arabia. Such innovations would help Vision 2030 achieve its objectives by curbing the negative impact of diabetes on the Saudi people. 

## Figures and Tables

**Table 1:** Sociodemographic characteristics of the participants (*n* = 392).

Variable	Number	Percentage (%)
**Gender**		
Male	180	45.9
Female	212	54.1
**Age**		
<30 years	34	8.6
31–50 years	278	70.9
>50 years	80	20.5
**Marital status**		
Single	32	8.2
Married	310	79.1
Other	50	12.7
**Level of Education**		
No education	35	8.9
Primary	178	45.4
Secondary	110	28.1
Tertiary/University	69	17.6
**Area of living**		
Rural	290	73.9
Urban	102	26.1
**Monthly family income**		
<5000 SR	143	36.5
5001–10,000 SR	181	46.2
10,001–20,000 SR	45	11.5
>20,000 SR	23	5.8
**Occupation**		
Business or private	156	39.8
Government employee	123	31.4
Housewife	67	17.1
Other	46	11.7

**Table 2:** Binary logistic regression to identify the significant structural factors that predict satisfaction with the services provided by diabetes clinics (*n* = 392).

Structural Factor	Satisfied with the Overall Services Provided by the CENTER OR (95% CI)
**Distance to the Center**	
More than 5 km	Reference
2–5 km	1.45 (0.91–2.77) ns
Less than 2 km	1.98 (1.08–3.44) *
**Average waiting time**	
More than 4 h	Reference
2–4 h	1.08 (0.76–1.78) ns
Less than 2 h	1.56 (0.81–2.13) ns
**Transport facilities**	
Not satisfactory	Reference
Satisfactory	1.67 (1.11–2.78) *
**Availability of other services** **(Counselling/Health education etc.)**	
Not satisfactory	Reference
Satisfactory	2.03 (1.12–4.04) *
**Service quality of doctors**	
Not satisfactory	Reference
Satisfactory	2.54 (1.21–3.98) *
**Service quality of Nurses**	
Not satisfactory	Reference
Satisfactory	1.04 (0.67–1.67) ns
**Service quality of Pharmacists**	
Not satisfactory	Reference
Satisfactory	1.66 (1.06–2.17) *
**Service quality of Lab Technicians**	
Not satisfactory	Reference
Satisfactory	1.56 (1.03–2.44) *

* *p* < 0.05; ns—not significant.

## Data Availability

The datasets generated and/or analyzed in the current study are available from the corresponding author and will be provided on reasonable request.
